# New Appliances and Things Medical

**Published:** 1897-01-30

**Authors:** 


### NEW APPLIANCES AND THINGS MEDICAL.

[We shall be glad to receive, at our Office, 28 & 29, Southampton Street, Strand, London, W.C., from the manufacturers, specimens of all new preparations and appliances which may be brought out from time to time.]

### PALATINOIDS OF GUAIACOL AND CREASOTE.

(Oppenheimer, Son, and Co., Worship Street, London, E.C.)

We have lately received samples of the above. The guaiacol palatinoids contain two minims each, they are beautifully made, and render the administration of this valuable drug a matter of great simplicity. In phthisis any form of guaiacum is often of the greatest advantage to the patient, and in many septic conditions it has afforded wonderfully good results; but in no form is it better tolerated or more efficacious than as guaiacol, and now that it can be administered in the pleasant form of palatinoids its use is likely to extend rapidly. The palatinoids of creasote also contain a dose of two minims. They are guaranteed not corrosive, and absolutely free from cresols. The value of creasote in phthisis and other lung troubles with fœtid expectoration, as well as in many gastric conditions, is so well known that this elegant method of administration will be welcomed both by physician and patient.

